# December Thoughts

**Published:** 1896-12

**Authors:** 


### Editorial.

### DECEMBER THOUGHTS.

THE reflections that come to all, as nature robes herself in the garment of approaching winter, are of the past year, its work and the estimate which must be placed upon it. Retrospection is not always profitable, yet it must ever be suggestive to the thoughtful mind. It means a personal criticism, an accounting, so to speak, for all the omissions and commissions that have been for the good or ill of the individual or the community with which he is surrounded. To the professional laborer it means more than this, for it should recall to him the sense of the responsibility that belongs to him as a part of the great work, and the question should be ever present, Has the labor allotted me been performed with an ever-present aim for the good of my calling, and with a loftier aspiration for the greatest benefit possible to those intrusted to my care?

That which is true of the individual is equally true of the profession represented. The twelve months should indicate progress in many directions, for, if this be not apparent, it means a retrograde, and this is not in harmony with the spirit of the age.

A review of the work performed in dentistry, in the period named, is not possible here, nor would it be desirable, for each worker can decide for himself whether this labor has been satisfactory or otherwise. That the year 1896 has not been a barren year must be conceded, but that it has been a prolific one, either in the professional or greater world around us, cannot be admitted.

Dentistry, as far as the United States is concerned, has been passing through a crucial period. It would seem, as we read its history for the past year, to have been, at times, under a cloud, and yet when the work is examined in detail, it compares very favorably with other years in the past. While this is true, there has been an apparent sluggishness, a lack of vitality, disclosed in professional work not altogether encouraging. Why this should have been more manifest this year it is difficult to determine, but, if it be true, it indicates not so much a lack of interest in the work, as it shows a feeling to exist that old methods of procedure have had their day and the active minds are looking for other avenues and other means whereby the profession may be advanced. It is certainly true that the tendency has been rather towards a selfish isolation, a feeling that dentistry has reached a period when it does not require the united efforts of every individual, but can be left to itself, or, in other words, to those who have grown old in the service. This idea, if entertained, is a wrong one, for it is only by the admixture of new blood with the old that the body can maintain a healthful activity. This retrogressive tendency has been manifested in an indifference to organized effort, perhaps more so here than in any other direction. Meetings have not exhibited the vitality of the past, in fact, have at times dragged heavily. Papers covering new lines of investigation have not been plentiful in our local, State, or national organizations, indeed originality has been sadly lacking in the work of the year. While this should not be a depressing fact it is not a pleasant reflection. The consolation, if any is to be found, must rest in the conviction that mind as well as nature must lie fallow at certain periods that in the end better results may be attained. It is hoped that the present lukewarmness may be the precursor of that greater awakening yet to come.

Three years yet remain to us of the present century, and dentistry should see to it that the end be worthy of the great era, perhaps the greatest in the world's advance in civilization.

It must not be understood that, in the opinion of the writer, the year nearing its close has been wholly unproductive. In many directions, professionally speaking, it has merited much honor. Some things have been accomplished which give hopes of important results for humanity, notably the final demonstration, practically begun last year, of electrical osmosis. This seems to promise to do for dentistry what anæsthesia did for general surgery, and if this prove true, it marks an epoch in its life hardly second to anything that has preceded it.

The silent reaper has been active during the past twelve months, and a number of those who have added interest and strength to our calling, have joined the host of the immortals. This, while it weakens the band of the faithful, must not be a source of anxiety, such has ever been and will ever be in this changing life. It, however, should be the incentive to more active work. The new dentist coming forward from college training, with his more thorough scientific methods, is the man of the future, and to these the right hand of fellowship must be given that the gaps in the ranks may be worthily filled, and that the eternal and ever-ascending spiral may be towards greater perfection in every branch of our work.

The year has given its share of new books. These have generally been above the average and indicate a decided growth in that direction. Books may truly be said to be the measure of professional depth, and, if this be true, the signs are hopeful for the future, for the supply indicates a higher demand than has existed in the past. The periodical literature has been steadily improving, if we except those journals that feed principally upon the crumbs falling from other tables, but even these are manifesting a desire to rise above their origin, that of trade, and it is to be hoped they will eventually succeed.

The colleges have steadily advanced their standards, and with new buildings and broader curricula there is abundant hope that the coming dentist will be thoroughly grounded in the work of the profession.

Thus, as the winter falls with his icy mantel upon us, the prospective warmth of the spring of the new year gives confident anticipations that the budding of leaves will show that a newer and more productive activity is for us in the future, and may we all rise to meet it with an energy worthy the life of 1897, which is yet to be.

